# Benign Recurrent Intrahepatic Cholestasis in Pregnancy: Fetal Death at 36 Weeks of Gestation

**DOI:** 10.1155/2021/5086846

**Published:** 2021-09-06

**Authors:** Mariam Ayyash, Nicolina Smith, Madhurima Keerthy, Ashina Singh, Majid Shaman

**Affiliations:** ^1^Department of Obstetrics and Gynecology, Henry Ford Health System, Detroit, Michigan, USA; ^2^Department of Gastroenterology and Hepatology, Henry Ford Health System, Detroit, Michigan, USA; ^3^Division of Maternal and Fetal Medicine, Department of Obstetrics and Gynecology, Henry Ford Health System, Detroit, Michigan, USA

## Abstract

**Introduction:**

Benign recurrent intrahepatic cholestasis is a rare hepatologic disorder characterized by recurrent, self-limited episodes of severe pruritus, jaundice, and elevated bile acids. While there are guidelines for the management of intrahepatic cholestasis of pregnancy, the literature regarding benign recurrent intrahepatic cholestasis and pregnancy is limited.

**Case:**

A 29-year-old G1P0 woman, with history of liver toxicity, had elevated total serum bile acid levels and liver enzymes documented at 8 weeks of gestation and throughout her pregnancy. She had a reactive nonstress test just 3 days prior to her induction. Fetal demise was noted when she presented at 36 weeks for her induction.

**Conclusion:**

We recommend that women with elevated total serum bile acid early in pregnancy due to a separate entity relative to intrahepatic cholestasis of pregnancy be managed in a more individualized approach.

## 1. Introduction

Benign recurrent intrahepatic cholestasis (BRIC) was first described in 1959 [1]. It can present any time in life, from infancy to late adulthood, and is characterized by recurrent self-limited episodes of severe pruritus and jaundice that can last from several weeks to months [2]. BRIC does not lead to progressive liver dysfunction and cirrhosis, but the symptoms associated with each attack may be associated with significant morbidity [2]. The pathophysiology of BRIC is poorly understood, and treatment remains empiric, primarily aimed at symptom relief [3]. The literature addressing BRIC during pregnancy is limited, and many patients with an abnormal liver profile and total serum bile acids (TSBA) are managed as having intrahepatic cholestasis of pregnancy (ICP).

ICP is generally characterized by pruritus and an elevation in liver enzymes and/or TSBA concentrations, and importantly, it typically develops in the second or third trimester [4, 5]. The etiology of ICP is not fully understood; it is associated with adverse fetal outcomes such as increased risk of preterm delivery, intrapartum fetal heart rate abnormalities, meconium-stained fluid, neonatal respiratory distress syndrome, and fetal death [5, 6]. The severity of ICP ranges from mild itching during pregnancy to fetal death [7]. Given the risk of fetal death within the context of ICP, the Society for Maternal-Fetal Medicine recommends induction of labor for women with ICP in the late preterm/early term period, specifically between 36 weeks and 39 weeks if TSBA is under 100 *μ*mol/L and at 36 weeks if TSBA is more than or equal to 100 *μ*mol/L [8]. Because such recommendations are specifically for ICP, there are no particular guidelines for labor induction within the setting of BRIC or other cholestatic liver disorders, when bile acid levels may be elevated within the first trimester.

We present the case of a patient with presumed BRIC for whom we followed the guidelines for women with ICP for delivery with induction at 36 weeks. However, upon presenting at her 36-week gestation mark for induction of labor, the patient had fetal death.

## 2. Case Presentation

A 29-year-old gravida 1 woman presented at 6 weeks and 2 days of gestational age for prenatal care. She had a history of liver toxicity that occurred in 2009 and again in 2014, which was thought to be related to drug-induced liver injury caused by oral contraceptive pills. At the time of her liver toxicity episodes, she had had an extensive infectious and autoimmune workup, which included testing for acute and chronic hepatitis A, hepatitis E, hepatitis B, hepatitis C, Epstein-Barr virus, cytomegalovirus, and human herpesvirus, all of which were negative. A liver biopsy was taken in 2014 which was consistent with possible drug-induced cholestasis. At the beginning of her pregnancy, she started having symptoms of pruritus, dark urine, and pale stool. She was followed by the hospital's monthly maternal critical care conference meetings from the beginning of her pregnancy because of her cholestasis diagnosis, elevated liver enzymes, and initial elevated TSBA levels of 215 *μ*mol/L at 8 weeks and 3 days of gestation.

**Figure 1:**
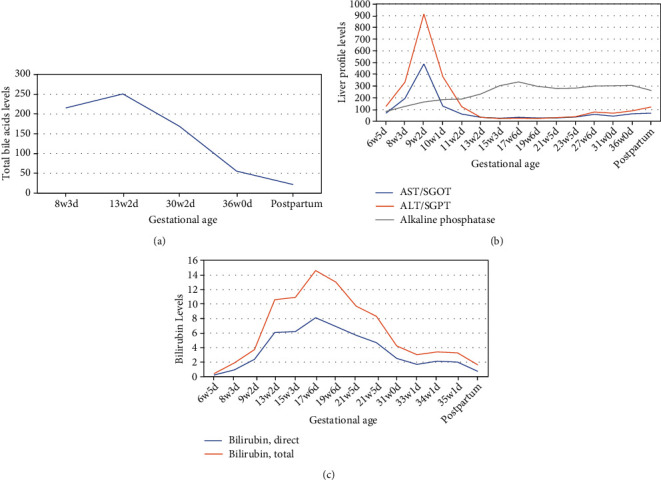
(a) Trend in bile acid levels. (b) Trend in liver profile values. (c) Trend in bilirubin levels (note: “postpartum” refers to 12 days following delivery).

She was being followed by hepatology experts and was initially started on ursodeoxycholic acid (UDCA) 300 mg 3 times a day, which was subsequently increased to 600 mg 2 times a day in her first trimester. She reported mild improvement in her symptoms after she was started on ursodiol. Her liver enzymes, which had been in a mixed hepatocellular and cholestatic pattern, improved after starting the UDCA. An underlying genetic cause within the differential diagnosis was suspected as the culprit of her clinical presentation, especially because of the patient's previous recurrent cholestatic episodes before her pregnancy. The follow-up plan during her pregnancy included liver profile and bile acid monitoring every 2 to 4 weeks (Figures 1(a) and 1(b)), weekly bilirubin checks (Figure 1(c)), serial growth ultrasounds every 4 weeks, nonstress tests twice weekly starting at 32 weeks of gestation, and ultimately delivery via induction of labor at 36 to 37 weeks and pending maternal and fetal status. Her growth ultrasounds were within normal limits with the last growth done at 32 weeks of gestation. Because of her increasing TSBA levels, the patient's UDCA dose was increased to 600 mg 3 times daily (15 mg/kg/day) (Figure 1). The plan was then to schedule induction of labor at 36 weeks with administration of 2 doses of betamethasone before her scheduled induction. When she presented for her betamethasone, she had a reactive nonstress test and received her first dose of betamethasone followed by a second dose the next day.

She arrived at 36 weeks and 0 days for induction of labor. She reported having contractions and leakage of yellow-brown fluid, with decreased fetal movement. The patient's cervix was 4 cm dilated with no evidence of prelabor preterm rupture of the membrane. No fetal heart tones were noted, and fetal death was confirmed upon evaluation by 2 healthcare providers. The patient was induced at that time with oxytocin and vaginally delivered a male newborn a few hours after the start of her induction. The newborn had normal anatomy. The patient had fetal death laboratory workup that included assessment of Kleihauer-Betke fetal hemoglobin, complete blood count, thyroid-stimulating hormone, and lactate dehydrogenase levels, in which all were within the reference range, as well as testing for the presence of toxoplasmosis, parvovirus, and antiphospholipid syndrome, all of which were negative. Her liver enzymes remained elevated (Figure 1(b)). A karyotype was performed on the newborn and showed a normal male karyotype. The patient underwent genetic testing after delivery, and her result showed a heterozygous variant of *ABCB11*, which is one of several recessive alleles associated with BRIC.

## 3. Discussion

Our patient had a unique presentation of cholestasis during pregnancy, which was later confirmed as BRIC. Her TSBA levels had remained above 200 *μ*mol/L after the first trimester and went under 100 *μ*mol/L only when she presented with the fetal death at the time of her scheduled labor induction. At that time, though, the value remained above 40 *μ*mol/L, a cutoff value that traditionally defines severe ICP and is associated with increased incidence of adverse fetal outcomes [5]. The mechanism by which elevated TBSA levels lead to adverse pregnancy outcomes, including fetal death, has not been well established. While there is an increased rate of fetal death with higher-than-normal TSBA levels, the literature suggests that the risk of fetal demise is significantly higher for women with TSBA levels specifically 100 *μ*mol/L or higher [9, 10]. It is also worth noting that the impact of the timing of TSBA elevation on pregnancy outcomes, particularly when it occurs in the first trimester, has not been well explored in the literature, probably because there is a paucity of knowledge on the natural history of BRIC and ICP when bile acids are elevated early in pregnancy. In patients who have particularly severe TSBA elevation early in pregnancy, individualized plans need to be made for monitoring the fetus, and consideration should be given to the timing of induction possibly before 36 weeks and should be based on specific TSBA values.

The literature has cited 3 case reports describing pregnancy outcomes of women with the ICP onset in the first trimester due to elevation in bile acids then [11–13]. While these cases were labeled as first trimester ICP, they could very well have been BRIC, given the recurrence of cholestasis episodes outside of pregnancy and the early onset of TBSA elevation in the first trimester. In fact, it is thought that BRIC among most women is underdiagnosed because the two most common features of BRIC, pruritus and jaundice, are misattributed to either pregnancy or to the use of oral contraceptive pills [14].

Our patient was treated per the ICP guidelines, with appropriate monitoring and a plan for labor induction at 36 weeks of gestation, but ultimately had fetal death. Our patient's presentation of cholestasis was unique, as her TSBA levels were over 200 *μ*mol/L in her first trimester and then gradually decreased to the 100 s *μ*mol/L range throughout the remainder of her pregnancy. At the time of labor induction, her TSBA level was at 56 *μ*mol/L, well under 100 *μ*mol/L; however, it is still within the relatively severe range during pregnancy, and no literature has evaluated the impact on pregnancy outcomes of such TSBA elevations in the first trimester. The current Society for Maternal-Fetal Medicine guidelines recommend induction at 36 weeks of gestation for women with ICP and TSBA greater than or equal to 100 *μ*mol/L [8]. The American College of Obstetricians and Gynecologists has also suggested that delivery before 36 weeks of gestation may be indicated depending on laboratory and clinical circumstances; however, clear-cut timeframes or criteria were not defined [15]. Often, TSBA levels are not assessed in cases of cholestasis at all because of lengthy lab resulting times, which can take anywhere between 2 and 6 days. While TSBA enzymatic test processing can be done within less than two hours, due to the low number of samples requested to be processed, technicians often wait to add more samples to cover the processing cost. This often results in the few days of delays in reporting results. Designing individual tests or evaluating TSBAs on a routine basis can help resolve such processing concern. In fact, due to its association with adverse pregnancy outcomes, we suggest that TSBA be routinely ordered for women with a history of liver/biliary disease in the first trimester as elevated TSBA results can influence management plans throughout pregnancies. In situations where a patient's TSBA levels are known and an alternative diagnosis to ICP is suspected because of TSBA elevation in the first trimester (particularly with levels over 100 *μ*mol/L), it is important to take these circumstances into account when considering the induction timeframe for pregnant women with ICP in the setting of likely a coexisting cholestatic disease. Little is known about the natural history of women with cholestatic disorders that are separate from ICP, and because reliable research on how these women fare during pregnancy is lacking, an individualized approach to care should be taken. Additionally, it is recommended that in cases where abnormally high TSBA levels occur within a setting of pruritus and elevated transaminase levels early in pregnancy, patients should be sent for genetic testing. In fact, this is recommended in the newly outlined practice guidelines on reproductive health in liver disease from the American Association for the Study of Liver Diseases [16].

We presented the case of a woman with presumed BRIC who was treated per the current ICP guidelines and yet had fetal death at 36 weeks of gestation, on the day of her planned labor induction. We recommend that women in early pregnancy who have elevated TSBA levels that may be the result of a cholestatic condition other than ICP should not be restricted to the current treatment and delivery guidelines that are specific for ICP. Regardless of whether they have BRIC or another cholestatic liver disorder, these women should be cared for with a more individualized approach: better established guidelines regarding the standardization and frequency of bile acid testing, antepartum testing, and patient education are needed, and more attention should be given to patients with a history of fetal death and cholestasis, with considerations such as antepartum admission with regular nonstress testing monitoring close to the timing of delivery, with induction earlier than the recommended 36 weeks. Lastly, we hope that a national registry can be established to improve the study of cholestatic disorders that are distinct from ICP to better guide treatment plans for these patients.

## Data Availability

The case data used to support the findings of this study are included within the article.
